# Effect of Nano-CSH and sodium sulfate on strength and durability of non-steam-curing high volume fly ash concrete

**DOI:** 10.1371/journal.pone.0326140

**Published:** 2025-07-10

**Authors:** Yongkang Du, Bitao Zhang, Dong Liu, Haoqian Wang, Yanyan Hu

**Affiliations:** 1 Shaanxi Building Materials Technology Group Co., Ltd, Xi’an, China; 2 Huangling Circular Ecological New Building Materials Co., Ltd, Yan’an, China; 3 College of Materials Science and Engineering, Xi’an University of Architecture and Technology, Xi’an, China.; Mirpur University of Science and Technology, PAKISTAN

## Abstract

In this study, a novel nanomaterial early strength agent, CSH nano-crystal nucleus (NCSH), was used to compare with the conventional early strength agent, sodium sulfate (Na_2_SO_4_), to deal with the problem of insufficient early strength development of high volume fly ash concrete. The effects of NCSH, Na_2_SO_4_, and their combined action on the strength development, water absorption performance, and durability performance (impermeability, frost resistance, and carbonation) were investigated. The research results show that: the maximum strength of the concrete with Na_2_SO_4_ was only 12.9 MPa at 12 hours, which could not meet the requirement of demoulding. At 28 days, the strength had significantly decreased, and the larger the dosage, the more it had decreased. Using NCSH or a mix of NCSH and Na_2_SO_4_ allowed the concrete to attain the necessary strength for demoulding at 12 hours, and none of them showed any reduction at 28 days. The concrete durability test results show that: Na_2_SO_4_ decreased the impermeability, frost resistance and carbonation resistance of concrete, while NCSH improved above properties of concrete significantly, and the improvement of frost resistance and carbonation resistance when combined Na_2_SO_4_ and NCSH was not as good as that of NCSH alone. In addition, either NCSH or the combination of CSH and Na_2_SO_4_ could make the concrete reach the frost resistance level of F200, and the concrete with appropriate dose of NCSH can still maintain the low mass loss rate and high relative dynamic elastic modulus after 200 freeze-thaw cycles; while the concrete with Na_2_SO_4_ did not reach F200.

## 1. Introduction

In order to save production cost, a large amount of fly ash will be mixed into concrete products, but according to the research, it is found that fly ash will lead to slow development of the early strength of the concrete [1–3], concrete can not reach the demoulding strength in time, the production efficiency is low, and the economic cost is increased [4]. Therefore, many factories use steam curing to rapidly improve the early strength of concrete. However, with the continuous deepening of the study, researchers found that, due to the different thermal expansion coefficients of concrete elements [5,6], the vaporization expansion of water tends to cause internal expansion and cracking of concrete, resulting in microcracks, which can cause damage to the hardened concrete structure [3,6,7]. In addition, the rate of cement hydration reaction is too fast during high temperature steam curing, but the transfer of hydration products elsewhere is relatively slow, resulting in rough and loose hydration products, which are not conducive to filling capillary pores [8,9]. It has even been shown that [1,10] the rapidly generated hydration products cannot diffuse in time and will gather around the unhydrated cement particles, gradually forming dense inclusions that prevent further hydration of the cement particles and eventually cause the concrete strength development to become slow [3]. And the high energy consumption of steam curing is not in line with the international trend of green and sustainable development, the current direction of research is to allow concrete to quickly increase strength through the use of early-strength agents.

The commonly used early strength agents are calcium sulfate, sodium sulfate (Na_2_SO_4_), triethanolamine, calcium formate, etc., which mainly rely on the reaction with cement minerals C_3_A and C_4_AF to generate complex compounds that can promote coagulation to achieve the goal of rapidly increasing strength. However, traditional early strength agents have adverse effects on the long-term performance of concrete. For example, in the single mixing of sulphate system early strength agent, SO4^2-^ tends to form calcite precipitates in the concrete, which increases the pore stresses and ultimately leads to plastic deformation of the concrete, resulting in the destruction of the concrete structure [11–13]. And when the dosage of organic early strength agents such as triethanolamine and calcium formate [14,15] is large, it will slow down the setting time of concrete and greatly reduce the construction efficiency.

With the rapid development of nanomaterials bringing technological innovation to the traditional concrete industry [16,17], CSH nano-crystal nucleus (NCSH) was introduced into concrete for research and is considered one of the most promising concrete admixtures [18–20]. NCSH play the role of nucleation seeds in cementitious [21–23], which can reduce the nucleation potential energy in the liquid phase during pre-hydration of cement thereby accelerating the precipitation and enrichment of hydration products and accelerating the hydration reaction process of cement [24,25]; they also play the role of microfilling, which can optimize the pore characteristics inside the matrix and refine the pore size structure [26,27], thus promoting the development of mechanical properties of concrete as well as improving its durability [28–30]. Several studies [31,32] reported the effect of NCSH on the hydration reaction of cement, and the addition of NCSH to cement accelerated the hydration reaction of cement minerals, reduced the volume fraction of harmful pores, and improved the early compressive strength of cementitious. It was also found that [33] when NCSH and sodium sulfate (Na_2_SO_4_) were mixed into concrete, they could enhance the early compressive strength, and when 0.5% of NCSH and 1.0% of Na_2_SO_4_ were combined together, the 24h compressive strength of cement was increased by 180%, which was much greater than the strength increase of NCSH and Na_2_SO_4_ alone. This shows that there may be a synergistic effect of NCSH and traditional early strength agents [24,34], but the effect on concrete performance when the two are compounded has not been reported.

In this study, to address the problem of insufficient early strength development and poor durability of non-steam-curing high volume fly ash concrete, NCSH was introduced to study the effect of nano early strength agent on the early strength development, and the traditional early strength agent Na_2_SO_4_ was selected for a comparative study, to further investigate the effect of compound NCSH and Na_2_SO_4_ on the concrete performance. This may help to replace the traditional industrial techniques to promote the early strength development of precast concrete, enable the demoulding of precast concrete under steam-free conditions as soon as possible, and save energy consumption at the same time, which has strong practical engineering significance.

## 2. Experimental program

### 2.1. Materials

**Fig 1:**
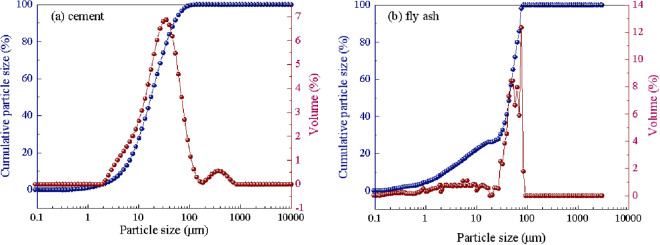
Particle size distribution of raw materials. (a) cement (b) fly ash.

The primary chemical composition and physical properties of the P.O. 42.5 cement and fly ash used in this study are shown in Tables 1 and 2, and the particle size distribution is shown in Fig 1. Table 1 lists the chemical composition of cement and FA. Table 2 lists the physical properties of cement, and the particle size distribution is shown in Fig 1. The solid content-concentration of polycarboxylate superplasticizer is 10% and the water-reducing rate is 30%. The polycarboxylic acid water reducer with 30.0% of water reducing rate was used. Gravel (particle size: 5 ~ 30 mm) and river sand (fineness modulus: 2.8) are the aggregates. “VIVID-300 nano-crystal nucleus” is provided by Shanghai Sanrui Polymer Material Co., Ltd. with the appearance of white, inodorous and stable suspension, solid content- concentration of 20%, Na_2_SO_4_ early strength agent is colorless and transparent particles, purity ≥ 99%.

**Table 1:** Chemical compositions of cement and FA (wt, %).

Oxides Sample	CaO	SiO_2_	Al_2_O_3_	Fe_2_O_3_	MgO	SO_3_	Na_2_O	K_2_O	TiO_2_	LOI
Cement	62.56	18.17	4.75	3.32	2.22	3.28	0.43	1.28	0.36	1.43
FA	4.45	53.46	21.70	5.85	1.38	1.59	1.03	2.65	1.60	4.59

**Table 2:** Physical properties of cement.

Type	Specific surface area(m^2^/kg)	Setting time (min)		Flexural strength (MPa)		Compressive strength (MPa)	
		Initial setting	Final setting	3 days	28 days	3 days	28 days
P.O 42.5	345	205	270	5.7	8.4	22.3	47.5

### 2.2. Mixture proportions

The concrete mix proportions is shown in Table 3. Fresh concrete was placed into 100 × 100 × 100 mm molds, which were then placed in the standard curing room (temperature: 20°C, humidity ≥ 95%) and cured until the corresponding ages before being tested for strength.

**Table 3:** Mix proportion of concrete (kg/m^3^).

Series	W/B	Cement	FA	Gravel	Sand	NCSH/wwt%	SS/wwt%	PCE/ wt%
Blank	0.334	267	199	762	1052	–	–	2.40
NCSH-0.2	0.334	267	199	762	1052	0.2	–	2.22
NCSH-0.6	0.334	267	199	762	1052	0.6	–	2.03
NCSH-1.0	0.334	267	199	762	1052	1.0	–	1.85
NCSH-1.4	0.334	267	199	762	1052	1.4	–	1.48
SS-0.2	0.334	267	199	762	1052	–	0.2	2.59
SS-0.6	0.334	267	199	762	1052	–	0.6	2.68
SS-1.0	0.334	267	199	762	1052	–	1.0	2.77
SS-1.4	0.334	267	199	762	1052	–	1.4	3.14
NCSH-SS-0.4	0.334	267	199	762	1052	0.4	0.2	2.22
NCSH-SS-0.8	0.334	267	199	762	1052	0.8	0.2	2.03
NCSH-SS-1.2	0.334	267	199	762	1052	1.2	0.2	1.85

### 2.3. Test methods

The test of compressive strength was carried out following the Chinese standard GB/T 50081−2019. The concrete impermeability test was carried out according to the water penetration height method in the Chinese standard GB/T 50082−2019. The freeze-thaw test was carried out according to Chinese Standard GB/T 50082−2019, fast-freezing method. The frost resistance of the specimens was evaluated by determining two indicators of the relative dynamic elastic modulus and mass loss of the concrete during the test. The carbonation resistance test was carried out according to the GB/T 50082−2019 rapid carbonation test method, and the carbonation depth of each specimen was measured at 10 points according to the pre-defined measurement line, and the arithmetic mean was taken. Burker AXS D8 X-Ray Diffractometer (XRD) was used to analyze and characterize the composition and physical phase changes of hydration products. ZEISS Gemini 300 Scanning Electron Microscopy (SEM) was used to observe the microstructural changes of hydration products in specimens of corresponding ages; TGA/DSC2 HSS2 Thermogravimetric Analysis (TG-DTG) was used to analyze the thermal weight loss of hydration products.

## 3. Results and discussion

### 3.1. Compressive strength

**Fig 2:**
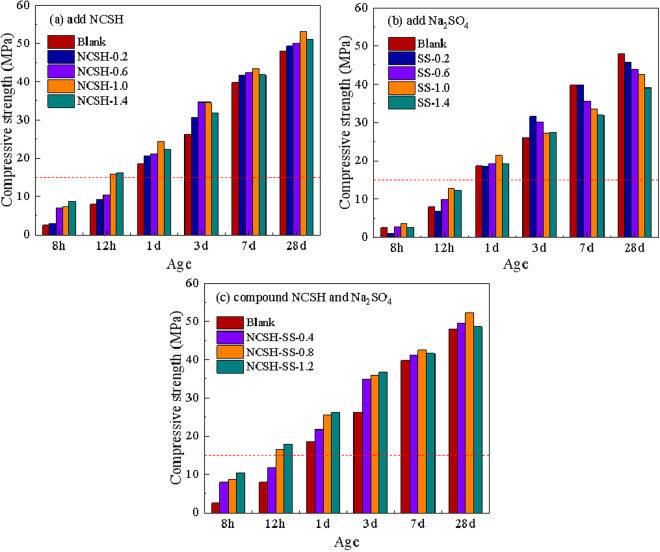
Compressive strength of concrete at different ages. (a) add NCSH(b) add Na_2_SO_4_(c) compound NCSH and Na_2_SO_4._

Fig 2 shows the strength development of the concrete with the addition of NCSH, Na_2_SO_4_, and a combination of the two. It is evident from Fig 2(a) that NCSH significantly impacts concrete strength, with the strongest increase in strength happens before 3 days and particularly within the first 12 hours. The long-term strength development is also significantly greater than that of the blank group. The appropriate dose of Na_2_SO_4_ has a certain enhancement effect on the early compressive strength of concrete, but the enhancement effect is not as obvious as that of NCSH, and it is very unfavorable to the long-term strength development. From Fig 2(c), it is clear that the combination of NCSH and Na_2_SO_4_ has the greatest impact on the strength improvement of concrete before 3 days, particularly on the strength improvement within 12 hours, and also ensures the development of long-term strength. With the exception of contains 0.4% of NCSH and 0.2% of Na_2_SO_4_, all other concrete strengths are sufficient for demoulding.

Compared with the addition of NCSH alone, the combination of NCSH and Na_2_SO_4_ at the same dosage has a more significant effect on the early strength of concrete, but the long-term strength development is not as good as that of adding NCSH alone, which may be due to the presence of Na_2_SO_4_ affecting the long-term strength development. Considering the long-term strength and economic cost, the addition of 0.8% NCSH and 0.2% Na_2_SO_4_ in the concrete at the same time can make the precast components demoulding as early as possible without steam curing and ensure the development of long-term strength. In practice, NCSH alone or in combination with Na_2_SO_4_ can be used according to the demand.

### 3.2. XRD test results

**Fig 3:**
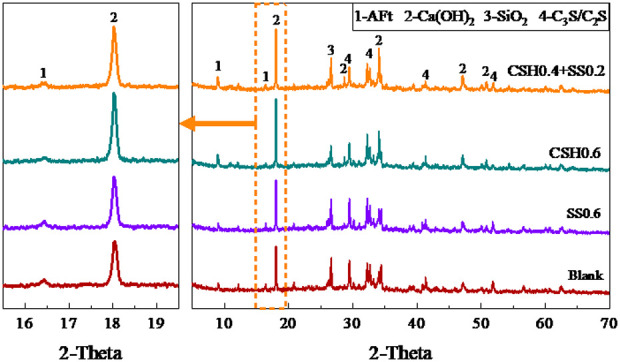
XRD pattern of hydration for 12 hours.

The XRD patterns of the specimens hydrated for 12 hours are shown in Fig 3. Compared with the blank group, no new matter was produced after the addition of NCSH or Na_2_SO_4_, and the positions of the diffraction peaks of the corresponding hydration products did not change. It is not difficult to find that the presence of NCSH significantly enhances the characteristic diffraction peak intensity of Ca(OH)_2_ and CaCO_3_, while decreasing the diffraction peak intensity of silicate minerals (C_3_S/C_2_S), indicating that the NCSH accelerate the hydration reaction of cement. The diffraction peak intensity of the silicate minerals is lower when the NCSH are compounded with Na_2_SO_4_, and a slight change in the diffraction peak intensity of Ca(OH)_2_ and ettringite in the range of 2θ = 16.0° to 18.5°. This may be due to the reaction between soluble sulfate and Ca(OH)_2_ and the further formation of ettringite [35–37]. This indicates that the NCSH compounded with Na_2_SO_4_ can promote the process of cement hydration reaction, especially in the early stage of hydration, thus promoting the formation of hydration products. Furthermore, the presence of NCSH was shown to reduce the strength of diffraction peaks of active SiO_2_ in fly ash, which might be attributed to NCSH’s accelerating impact at the early stage that activates the pozzolanic effect of fly ash. Only the diffraction peak of Ca(OH)_2_ was somewhat elevated with the addition of Na_2_SO_4_, while the intensity of the others did not change significantly.

### 3.3. SEM test results

**Fig 4:**
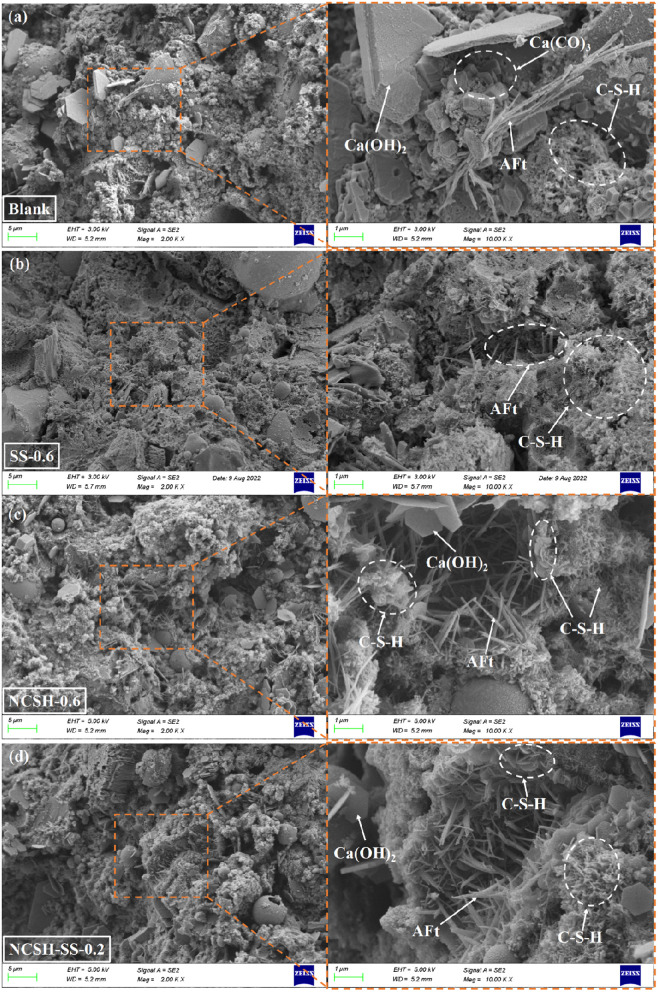
SEM image of hydration for 12h. (a) Blank(b) SS-0.6(c) NCSH-0.6(d) NCSH-SS-0.2.

Fig 4 shows the morphology of the hydration products of each group after 12h of hydration. According to Fig 4(a), only a very tiny quantity of hydration products such as C-S-H gel, ettringite, and Ca(OH)_2_ were dispersed around the blank specimen, and the structure was generally unstructured, with no definite contour of products. The C-S-H gel and ettringite somewhat increased with the addition of Na_2_SO_4_, and the AFt interspersed in the C-S-H gel progressively developed an interconnected structure, as shown in Fig 4(b).

From Fig 4(c) and 4(d), the addition of NCSH or compound NCSH and Na_2_SO_4_ resulted in a greater number of hydration products and a more regular and clear appearance of the products with a large number of C-S-H gels filling the pores. It follows that the presence of NCSH stimulates the hydration reaction of cement, provides more nucleation sites for hydration products, and reduces barriers to hydration product growth [19,38]. C-S-H gels nucleate and grow on the surface of cement minerals, but also more readily on the surface of CSH nanoparticles, which greatly accelerates the early hydration.

### 3.4. TG-DTG

**Fig 5:**
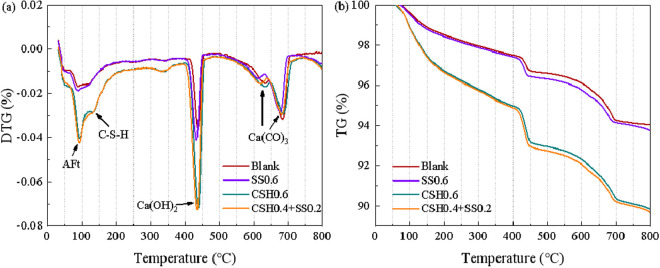
TG-DTG curve of the sample after 12 hours hydration. (a) DTG (b) TG.

The TG-DTG curves are shown in Fig 5. As shown in Fig 5(a), the first peak occurs between 100^o^C and 200^o^C, which is attributed to the dehydration of free water and bound water in the ettringite and C-S-H gels [39]. The second peak at around 450^o^C is the decomposition peak of Ca(OH)_2_, and the last peak at around 700^o^C corresponds to the decomposition of Ca(CO)_3_. As can be seen in Fig 5(b), in the temperature range corresponding to the dehydration peaks of ettringite and C-S-H gels and the decomposition peaks of Ca(OH)_2_, the mass losses in descending order are: Blank<0.6% Na_2_SO_4_<0.6% NCSH<0.4% NCSH and 0.2% Na_2_SO_4_. Higher mass loss rates were associated with larger amounts of the corresponding hydration products. The mass loss of samples containing NCSH was more severe, indicating that the presence of NCSH accelerated the hydration reaction, thus promoting the generation of C-S-H gels, ettringite and Ca(OH)_2_. When NCSH was used in combination with Na_2_SO_4_, the amount of hydration products generated was more abundant, which also confirmed the increase in early strength of concrete.

### 3.5. Water absorption of concrete

Water is an important factor affecting the durability of concrete, and the transport of water plays a crucial role in the deterioration of concrete structures, and the vast majority of durability problems are directly related to the presence of water and its transport.

**Fig 6:**
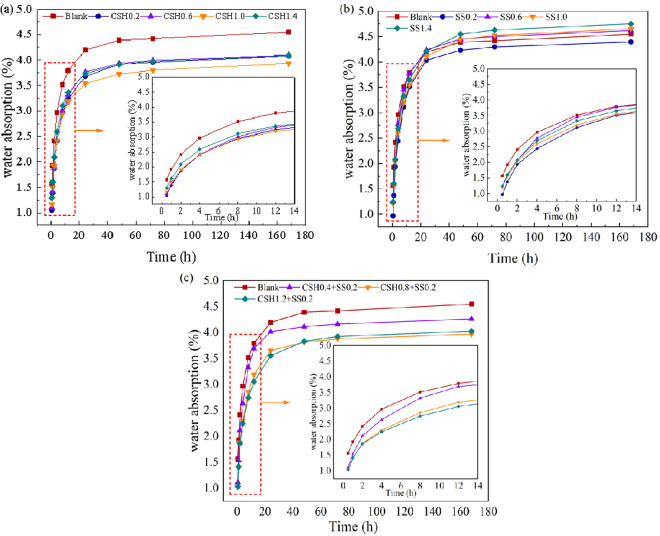
Concrete water absorption rate. (a) add NCSH; (b) add Na_2_SO_4_; (c) compound NCSH and Na_2_SO_4._

Fig 6 shows the variation of water absorption of concrete with time after the addition of different doses of NCSH, Na_2_SO_4_ and combination of both. As shown in Fig 6, with the increase of immersion time, the water absorption rate of concrete with NCSH is on the rise, and the water absorption rate is faster in the early stage, and starts to slow down with time, and the water absorption rate rises slightly until the specimen is saturated with water. Compared with the blank group, the addition of NCSH significantly reduced the water absorption rate and water absorption speed. While the concrete adding Na_2_SO_4_ had a larger water absorption rate, and after 24 hours of immersion, except for the concrete adding 0.2% Na_2_SO_4_, which was lower than the blank group, the water absorption rate under the rest of Na_2_SO_4_ dosing was higher than the blank group, and the water absorption increases with the increase of dosing. From the effect on the water absorption of concrete, the presence of Na_2_SO_4_ did not have a positive effect on the pore structure, and even deteriorated the pore structure [40]. Concrete’s water absorption rate and speed were decreased when NCSH and Na_2_SO_4_ were used simultaneously, and the lowest water absorption rate and speed were obtained by adding 0.8% NCSH and 0.2% Na_2_SO_4_. As shown in Fig6(c), the water absorption in the blank was 1.57% and 4.55% when the specimens were immersed in water for 0.5 hours and 7 days, respectively, while the water absorption with 0.8% NCSH and 0.2% Na_2_SO_4_ was 1.00% and 3.90%, respectively, which was significantly lower than that of the blank. But the water absorption after 7d was greater than when NCSH were used alone. This is because when NCSH combine with Na_2_SO_4_, the concrete has higher early strength and fewer surface defects, but the presence of Na_2_SO_4_ deteriorates the later mechanical properties, thus increasing the water absorption rate when the concrete is close to water absorption saturation.

### 3.6. Durability

#### 3.6.1. Impermeability.

**Fig 7:**
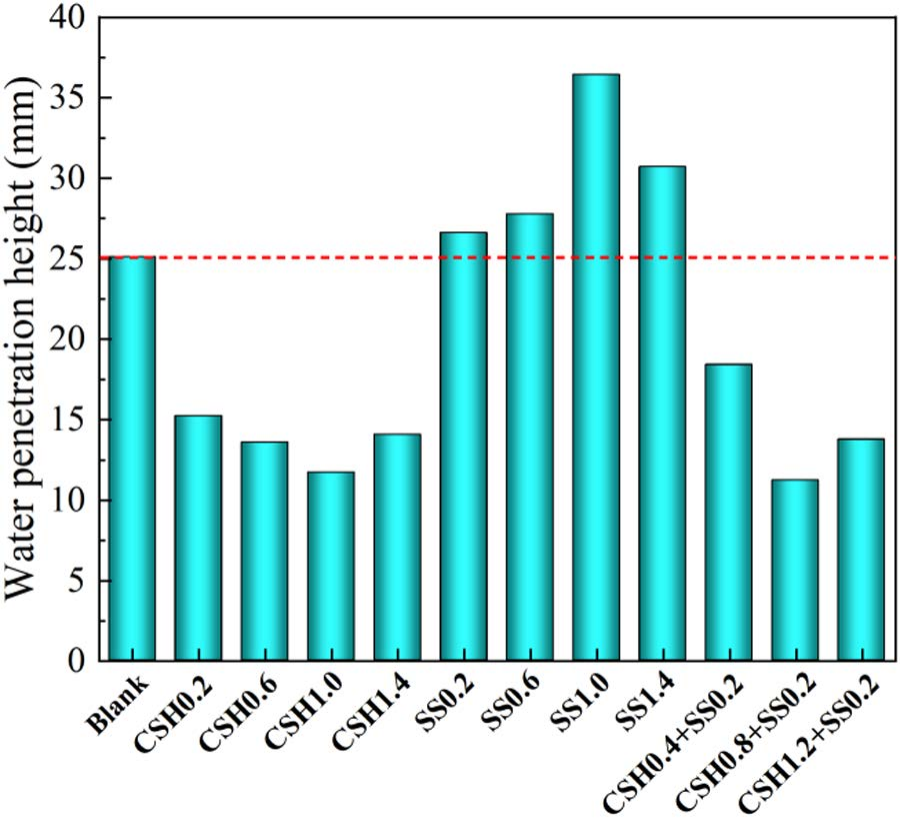
Water penetration height of concrete.

Fig 7 shows the effect of NCSH and Na_2_SO_4_ on the water penetration height of concrete. As shown in the figure, the addition of NCSH significantly reduced the water penetration height of concrete by a maximum of 53.4% compared to the blank group. While Na_2_SO_4_ increased the water penetration height, even 45.2% than the blank group at 1.0% dosing, which greatly reduced the impermeability of concrete. When NCSH were used in combination with Na_2_SO_4_, the concrete showed the best impermeability when 0.8% of NCSH and 0.2% of Na_2_SO_4_ were added.

The penetration resistance of concrete is related to the porosity as well as the pore characteristics; the lower the porosity of concrete and the fewer the connected holes, the better the penetration resistance [2,41]. The presence of NCSH improved the pore structure and increased the compactness of concrete. It has been shown that the addition of NCSH promotes the production of C-S-H gels and the hydration products grow more easily in the capillary pores, allowing them to be filled and refined. The possible reason for this is that the addition of NCSH caused the gradual transformation of connected pores into closed pores, reducing the channels for free water to enter the concrete interior [42]. In addition, there is a correlation between high compressive strength and low permeability because concrete impermeability is more sensitive to changes in pore and microcracks [10,43]. While Na_2_SO_4_ is partially adsorbed in the C-S-H gel after add into concrete, it is very easy to generate coarse-grained ettringite during cement hardening, which causes matrix expansion, deteriorates the pore structure, and increases the porosity of concrete, and the presence of sulfate promoted the dispersion of C-S-H gels and reduced the density of concrete.

#### 3.6.2. Frost resistance.

**Fig 8:**
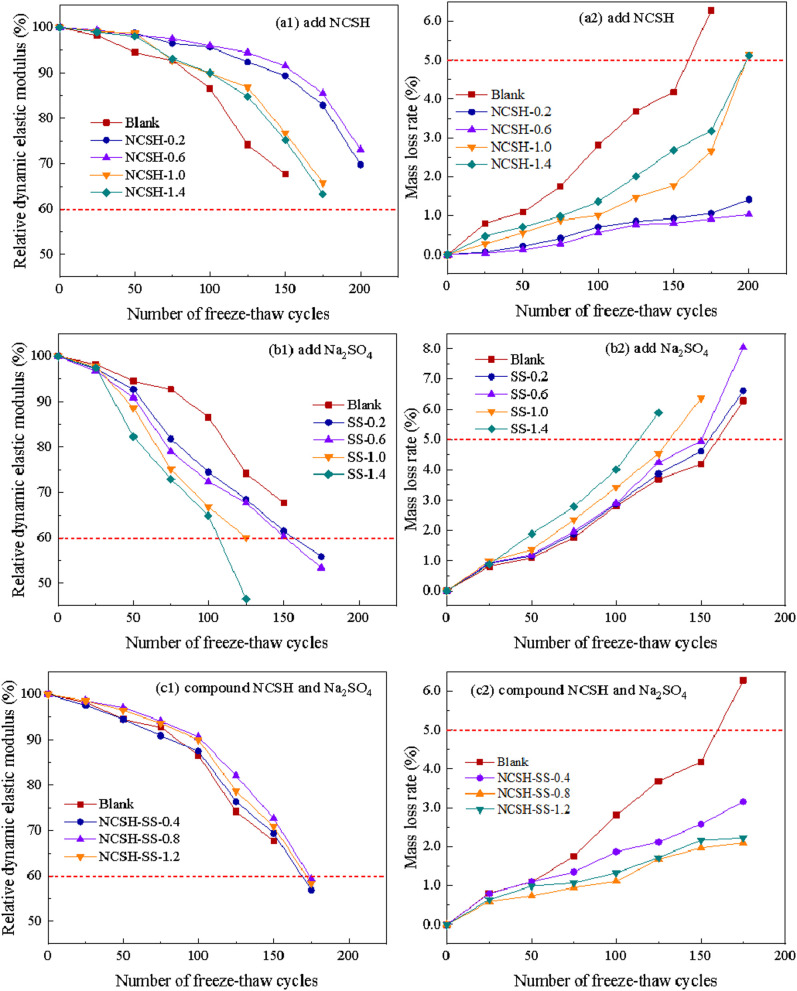
Relative dynamic elastic modulus and mass loss rate of concrete. (a1) add NCSH (a2) add NCSH (b1) add Na_2_SO_4_ (b2) add Na_2_SO_4_ (c1) compound NCSH and Na_2_SO_4_ (c2) compound NCSH and Na_2_SO_4._

Fig 8 shows the variation of the relative dynamic elastic modulus and mass loss of concrete. From the figure, it can be seen that the relative dynamic elastic modulus of all specimens gradually decreased and the mass loss increased with the increase of the number of freeze-thaw cycles, and the presence of NCSH slowed down the reduce of freezing resistance. As shown in Fig 8 (a1)(a2), the addition of NCSH alone had less effect on the freezing resistance before 150 freeze-thaw cycles, because the surface of concrete was less damaged or even not yet damaged at this stage. The maximum number of freeze-thaw cycles was 175 for NCSH dosing of 1.0% and 1.4%, while the blank groups were severely damaged at 175 cycles and the maximum number of freeze-thaw cycles was only 150. From Figure 8 (b1)(b2), it can be seen that the negative impact of Na_2_SO_4_ on the frost resistance of concrete is greater, the greater the content of Na_2_SO4, the faster the relative dynamic modulus of concrete decreases, the greater the mass loss, the maximum number of freeze-thaw cycles is basically 100–150 times. As shown in Figure 8(c1)(c2), when NCSH were used in combination with Na_2_SO_4_, the relative dynamic elastic modulus development trend was similar to that of the blank specimens, but the mass loss was smaller and slower, and the relative dynamic elastic modulus was slightly below 60% at 175 freeze-thaw cycles.

The reason why NCSH effectively improve the frost resistance of concrete is that the presence of NCSH fills the pore and improves its compactness, effectively hindering the entry of water and reducing the amount of free water that can occur inside the concrete [44]; on the other hand, NCSH improve the strength of concrete and increase the resistance to the expansion stress caused by water freezing. Furthermore, it is not difficult to find a decreasing trend in the improvement effect of NCSH on the frost resistance of concrete when the dosing of NCSH is too large, perhaps because the excessive nucleation sites increase the number of inter-gel phase interfaces and the increase of inter-gel pores leads to a decrease in the strength of concrete [36,45], thus weakening the resistance to frost damage.

#### 3.6.3. Carbonation resistance.

**Fig 9:**
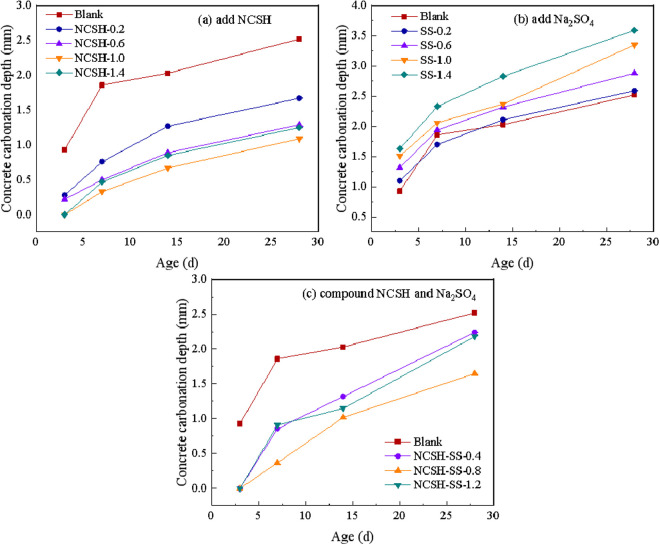
Carbonation depth of concrete. (a) add NCSH (b) add Na_2_SO_4_ (c) compound NCSH and Na_2_SO_4._

Fig 9 shows the variation curve of carbonation depth at different ages. As can be seen from the figure, the best anti-carbonation performance of concrete was obtained when 1.0% NCSH alone or combined 0.8% NCSH and 0.2% Na_2_SO_4_, while the depth of concrete carbonation was greater than that of blank specimens at almost all dosing levels when Na_2_SO_4_ was added, and the greater the dosing level, the worse the anti-carbonation performance. It was also found that there is a certain threshold value of NCSH dosing, beyond which the effect of improving the carbonation properties of concrete is diminished.

The filling effect and the acceleration of cement hydration of NCSH refine the pore structure of concrete and make it more compact, thus reducing the entry of aggressive gases into the concrete for damage. However, when the content of NCSH is too high, the presence of too many nucleation sites increases the number of interfaces between gels, resulting in a loose structure, which in turn promotes the entry of gas, so that the excessive amount of NCSH diminishes its effect on the improvement of concrete carbonation properties.

## 4. Conclusion

In this study, Na_2_SO_4_ was compared with NCSH to study the mechanism of NCSH on non-steam-curing concrete strength and durability (impermeability, frost resistance and carbonation resistance) under high volume fly ash, and further explored the effects on concrete properties when compounding Na_2_SO_4_ and NCSH. The findings of this study can be summarized as follows:

(1) NCSH can significantly accelerate the strength development, especially for the early strength, while Na_2_SO_4_ can only slightly promote it. Under standard curing conditions, NCSH or combined NCSH and Na_2_SO_4_ was able to make the concrete demoulding within 12 hours. But the long-term strength development was not as good as that of single admixture of NCSH when the two were used simultaneously.
(2) Na_2_SO_4_, NCSH or compounding NCSH and Na_2_SO_4_ can accelerate the hydration reaction of cement, resulting in a rapid increase in the early strength of concrete and without changing the type of hydration products. According to the XRD results, NCSH reduces the intensity of the fly ash’s active SiO_2_ diffraction peaks, which may be because NCSH activates the pozzolanic effect of fly ash. Whereas the addition of Na_2_SO_4_ increased the CH diffraction peaks, the intensity of other diffraction peaks did not change significantly, which implies that Na_2_SO_4_ has a detrimental effect on the long-term strength development and durability.
(3) The water absorption of concrete decreased with the increase of NCSH, while Na_2_SO_4_ increased the water absorption, and the improvement effect of NCSH was weakened when combined with Na_2_SO_4_. And Na_2_SO_4_ significantly reduces the impermeability, and the higher the dosage, the worse the impermeability of concrete.
(4) The simultaneous use of NCSH and Na_2_SO_4_ significantly increases the impermeability, frost resistance and carbonation resistance of concrete. The single use of NCSH can improve the impermeability and frost resistance, but the ability to improve carbonation resistance is weakened after mixing more than a certain amount. Na_2_SO_4_ has a greater negative effect on concrete durability, decreasing the relative dynamic elastic modulus, increasing mass loss, and increasing the depth of carbonation, but it slightly improves the impermeability at low dosages.

## Supporting information

S1 Data Compressive strength. (XLS)

S2 Data renamed_56233(TG). (XLS)
